# Factors influencing the development of osteoporosis in elderly patients with rheumatoid arthritis

**DOI:** 10.4314/ahs.v24i4.39

**Published:** 2024-12

**Authors:** Yanru Yang, Ying Chen, Li Zhang, Xiaofeng Yang, Jingquan Qiao

**Affiliations:** 1 Department of Rheumatology, Gansu Gem Flower Hospital, Lanzhou, China; 2 The First Clinical Medical College, Gansu University of Chinese Medicine, Lanzhou, China; 3 Department of Rheumatology, Lanzhou University Second Hospital, Lanzhou, China; 4 Department of Orthopaedic, Gansu Gem Flower Hospital, Lanzhou, China

**Keywords:** Elderly, rheumatoid arthritis, osteoporosis, influencing factors

## Abstract

**Background:**

To explore the influencing factors of osteoporosis (OP) in elderly patients with rheumatoid arthritis (RA).

**Methodology:**

A total of 145 elderly patients with RA were divided into comorbidity group (with OP) of 79 patients and RA group (without OP) of 66 patients. Demographic data and laboratory parameters were collected from patients. Demographic characteristics and laboratory parameters were compared between the two groups. Multiple influencing factors of OP in RA patients were analysed.

**Results:**

There were significant differences in age, BMI, primary disease duration, history of glucocorticoids (GC) administration, disease activity score in 28 joints (DAS-28), and Sharp score data between the two groups. There were significant differences in rheumatoid factor (RF), interleukin-27 (IL-27), procollagen I N-Terminal Propeptide (PINP), nuclear receptor of activator factor-κB ligand (RANKL), and 25-hydroxyvitamin D [25-hydroxyvitamin D, 25 (OH) D] data between the two groups (P < 0.05). Logistic analysis showed that age, primary disease duration, GC history, DAS-28, Sharp score, RANKL and 25 (OH) D were independent factors for OP in RA patients.

**Conclusion:**

The risk of OP in elderly RA patients is mainly related to age, primary disease duration, GC history, DAS-28, Sharp score, RANKL, and 25 (OH) D levels, and risk factors should be actively prevented.

## Introduction

Rheumatoid arthritis (RA) is an autoimmune disease typically characterized by erosive arthritis, and its prevalence continues to rise, with a heavy mental and physical burden on patients^1^. Osteoporosis (OP) is one of the common complications of RA and is a chronic progressive bone disease. Studies have reported^2^ that the prevalence of osteoporosis in RA patients decreased from 20% to 6% from 2007 to 2017. However, there remains a high risk of morbidity, and although the prevalence and risk of comorbidities continue to decline, their treatment is difficult and the therapeutic effect has not been effectively improved^3^,^4^. The study of factors influencing the occurrence of OP in elderly patients with RA is important to control disease progression and improve patient prognosis. Masamoto K et al study^5^ reported that bone mineral density (BMD) results were closely related to age, gender, disease activity score in 28 joints (DAS-28), erythrocyte sedimentation rate (ESR), and medication. At present, the influencing factors have not been completely unified in clinical practice, and more studies are still needed to demonstrate. Based on this, this study will analyze the influencing factors of OP in elderly RA patients in order to control the disease progression in RA patients.

## Patients and methods

### Patients

One hundred and forty-five elderly patients with RA who visited our hospital from March 2021 to June 2022 were selected and divided into comorbidity group (with OP) of 79 patients and RA group (without OP) of 66 patients according to whether they suffered from OP or not. This study was approved by the ethics committee of Gansu Gem Flower Hospital. Signed written informed consents were obtained from the patients and/or guardians.

The criteria for diagnosis was that: RA conforms to 2018 Chinese Guidelines for Diagnosis and Treatment of Rheumatoid Arthritis^6^: it shows morning stiffness, joint swelling and other clinical symptoms, and signs such as bone erosion are confirmed by laboratory (positive rheumatoid factor) and imaging X-ray. OP was in accordance with Chinese Guidelines for the Diagnosis and Treatment of Osteoporosis in the Elderly (2018)^7^: t-value of BMD ≤ -2.5 standard deviation (SD) and spinal deformation on imaging. Inclusion criteria set as: (1) Patients met the above diagnostic criteria for RA; (2) Patients with OP induced by RA, and aged ≥ 60 years; (3) Patients signed informed consent. Exclusion criteria involved that: (1) Patients with previous history of orthopedic surgery, systemic lupus erythematosus and other autoimmune diseases; (2) Patients combined kidney disease, heart disease, malignant tumors and other serious primary diseases; (3) Patients with incomplete clinical data.

### Methods

Demographic data and laboratory parameters were collected from patients, and demographic data included gender, age, BMI, primary disease duration, smoking history, drinking history, glucocorticoid (GC) medication history, hyperlipidemia, hypertension, diabetes, DAS-28, and Sharp score. DAS28 scale ≤ 2.6 indicates remission, 2.6 (excluding) -3.2 (including) indicated low mobility, 3.2 (excluding) -5.1 (including) indicated moderate mobility, and > 5.1 indicated high mobility. Sharp scoring criteria were as follows: proximal interphalangeal, metacarpophalangeal, and wrist bone erosion and joint space were assessed. A score of 0 indicated no erosion and 5 indicated extensive bone erosion and loss. The joint gap score is 0-4, with 0 points for no gap stenosis, 1 point for partial or very small area of stenosis, 2 points for extensive stenosis but >50% area of gap present, 3 points for extensive stenosis and <50% area of gap present, and 4 points for complete loss of joint gap, ankylosis or dislocation.

Laboratory parameters included rheumatoid factor (RF), C-reactive protein (CRP), interleukin-27 (IL-27), procollagen I N-Terminal Propeptide (PINP), receptor activator of factor-κB ligand (RANKL), and 25-hydroxyvitamin D [25-hydroxyvitamin D, 25 (OH) D]. Five milliliters of fasting venous blood was collected from the patients. RF and C-reactive protein (CRP) were detected by immunoturbidimetry (kit source: Wuhan Jingchuan Diagnostic Technology Co., Ltd., Wuhan, China). ESR was detected by erythrocyte sedimentation rate instrument (instrument source: Xunda ESR-30). RF was detected by rate nephelometry, and IL-27, PINP, RANKL, and 25 (OH) D were detected by enzyme-linked immunosorbent assay (kit source: Shanghai Enzyme-Linked Industrial Co., Ltd., Shanghai, China).

### Outcome Measures

(1) The demographic characteristics of RA patients between the two groups were compared; (2) the laboratory parameters of RA patients between the two groups were compared; (3) the multiple influencing factors of OP in RA patients were analysed.

### Statistical analysis

Data were included in Statistical Product and Service Solutions (SPSS) 23.0 software for analysis (IBM, Armonk, NY, USA). Measurement data following normal distribution in continuous variables were presented as mean ± standard deviation (^-^x±s), and t test was used for comparison; enumeration data were presented as χ^2^ test, and rate (%). logistic regression model was used to analyse multiple influencing factors of OP in RA patients, and (P < 0.05) was considered statistically significant.

## Results

### Demographic characteristics of RA patients were compared between the two groups

There were significant differences in age, BMI, primary disease duration, GC medication history, DAS-28 and Sharp score between the two groups (*P* < 0.05). See Table 1.

**Table 1:** Comparison of clinical characteristics between the two groups [n, x̅±s]

Demographic characteristics	Comorbid group (n = 79)	RA group (n = 66)	*χ^2^/t*	*P*
Gender (M/F)	29/50	23/43	0.054	0.816
Age (years)	75.14 ± 10.32	68.75 ± 9.24	3.893	< 0.001
BMI (kg/m^2^)	23.35 ± 2.14	24.67 ± 2.56	3.382	0.001
Primary disease duration (years)	11.23 ± 3.19	7.45 ± 2.56	7.761	< 0.001
Smoking history	18	10	1.345	0.246
Alcohol history	17	11	0.543	0.461
GC History	32	14	6.180	0.013
Hyperlipidemia	7	5	0.078	0.780
Hypertension	13	8	0.546	0.460
Diabetes	12	9	0.070	0.791
DAS-28 (points)	6.43 ± 1.25	5.17 ± 1.22	6.111	< 0.001
Sharp score (points)	84.35 ± 10.27	51.34 ± 9.43	20.001	< 0.001

### Comparation of the laboratory parameters of RA patients between the two groups

Patients in the co-morbid group had higher RF, IL-27 and RANKL than those in the RA group, and the differences were statistically significant (*P* < 0.05); patients in the co-morbid group had lower PINP and 25(OH)D than those in the RA group, and the differences were statistically significant (*P* < 0.05); while patients in the co-morbid group had slightly higher CRP than those in the RA group, and the differences were not statistically significant.. See Table 2.

**Table 2:** Comparison of laboratory parameters between the two groups of RA patients [x̅±s]

Laboratory Indicators	Comorbid group (n = 79)	RA group (n = 66)	*t*	*P*
RF (IU/mL)	91.23 ± 10.58	64.77 ± 10.21	15.237	< 0.001
CRP (mg/L)	17.44 ± 3.59	17.13 ± 3.58	0.519	0.605
IL-27 (pg/L)	57.64 ± 7.43	51.25 ± 6.48	5.463	< 0.001
PINP (ng/mL)	10.23 ± 2.48	14.32 ± 3.56	8.123	< 0.001
RANKL (pg/mL)	6.95 ± 1.32	6.24 ± 1.34	3.203	0.002
25 (OH) D (ng/mL)	12.35 ± 3.14	16.58 ± 3.29	7.904	< 0.001

### Analysis of multiple influencing factors of OP in RA patients

Logistic results showed that age, primary disease duration, GC administration history, DAS-28, Sharp score, RANKL, and 25 (OH) D were independent factors for OP in RA patients (*P* < 0.05), as shown in Table 3.

**Table 3:** Analysis of multiple influencing factors of OP in RA patients

Indicators	B	S.E.	Wald	*P*	OR (95% CI)
Age	0.779	0.181	14.378	< 0.001	1.654 (1.327-1.796)
BMI	0.400	0.235	2.899	0.089	1.491 (0.941-2.362)
Primary course	0.784	0.187	14.235	< 0.001	1.679 (1.314-1.843)
GC History	0.771	0.179	13.985	0.001	1.511 (0.360-1.727)
DAS-28	0.688	0.147	7.910	0.007	1.704 (0.675-1.833)
Sharp	0.635	0.130	6.608	0.010	1.398 (1.083-1.805)
RF	0.263	0.215	1.492	0.222	1.301 (0.853-1.983)
IL-27	0.237	0.237	1.145	0.475	1.059 (0.842-1.267)
PINP	0.235	0.223	1.237	0.235	1.247 (0.841-1.743)
RANKL	-0.578	0.453	7.382	0.014	0.815 (0.796-0.856)
25 (OH) D	0.737	0.323	5.187	0.023	2.089 (1.108-3.937)

B-value is the regression coefficient, SE is the standard error of B-value and Wald is the chi-squared value.

## Discussion

RA and OP share a common genetic background and pathogenic mechanism, and shared genomic regions include the GCKR and SERPINA1 genes; oxidative stress and inflammatory mechanisms are key pathogenic factors of RA, such as RA contributing to CRP elevation; while CRP and OP affected by RA show pleiotropic effects^8^. OP is characterized mainly by decreased BMD and trabecular microarchitecture deformation, and investigation of OP risk factors can be used as predictors of osteopenia, which is important for identifying patients at high risk of OP with RA comorbidity^9^. Kim et al.^10^ reported an increased risk of vertebral fractures with longer and higher doses of oral GC in RA patients.

The results of this study showed that there were significant differences in age, BMI, primary disease duration, GC medication history, DAS-28 and Sharp score data between the two groups (P < 0.05). There were significant differences in RF, IL-27, PINP, RANKL and 25 (OH) D data between the two groups (P < 0.05). Logistic analysis showed that age, primary disease duration, GC history, DAS-28, Sharp score, RANKL and 25 (OH) D were independent factors for OP in RA patients (P < 0.05). The reasons are as follows: (1) Age factors: As the body ages, there will be a significant decline in organ function and loss of bone mass, which in turn will lead to a decrease in bone density and the occurrence of OP; (2) Primary disease factors: the longer the duration of RA, the longer the duration of inflammation, the higher the risk of progression to chronic inflammation, the higher the degree of bone and joint erosion and activity destruction, bone resorption is much higher than the formation; (3) GC medication history factors: long-term use of GC may lead to increased osteoclast activity, bone formation decreased; (4) DAS-28 factors: DAS-28 score is closely related to the progression of RA, while the higher the score indicates that the more severe the patient's condition, the higher the disease activity, the higher the risk of OP complications in patients; (5) Sharp score factors: Sharp score factors: Sharp score is related to joint erosion and stenosis, the higher the imaging score indicates the presence of extensive stenosis, joint erosion, the higher the risk of OP; 7.25 (OH) D factor: 25 (OH) D level is closely related to the regulation of calcium and phosphorus metabolism in patients. When protein intake is insufficient, it will affect amino acid synthesis, and then have a certain impact on skeletal muscle quality and function.

## Conclusions

In summary, the risk of OP in elderly RA patients is mainly related to age, primary disease duration, GC medication history, DAS-28, Sharp score, RANKL, and 25 (OH) D levels, and risk factors should be actively prevented.

## Data Availability

The datasets used and analysed during the current study are available from the corresponding author on reasonable request.
